# Supplementary material for: Experimental observation of topological Z2 exciton-polaritons in transition metal dichalcogenide monolayers
Source: Nat Commun. 2021 Jul 20;12:4425. doi: 10.1038/s41467-021-24728-y (PMC8292485; doi:10.1038/s41467-021-24728-y)
Supplement: Supplementary file 1 — Supplementary Information [file 41467_2021_24728_MOESM1_ESM.pdf]

Supplementary Information for *Experimental observation of topological* Z₂ *exciton-polaritons in transition metal dichalcogenide monolayers*

Mengyao Li[1,2,3*], Ivan Sinev[4*], Fedor Benimetskiy[4], Tatyana Ivanova[4], Ekaterina Khestanova[4], Svetlana Kiriushechkina[1], Anton Vakulenko[1], Sriram Guddala[1,2], Maurice Skolnick[4,5], Vinod Menon[2,3], Dmitry Krizhanovskii[4,5], Andrea Alù[6,3,1], Anton Samusev[4], Alexander B. Khanikaev[1,2,3]

[1]Department of Electrical Engineering, City College of New York, New York, NY, USA

[2]Physics Department, City College of New York, New York, NY, USA

[3]Physics Program, Graduate Center of the City University of New York, New York, NY, USA

[4]Department of Physics and Engineering, ITMO University, Saint Petersburg, Russia

[5]Department of Physics and Astronomy, University of Sheffield, Sheffield S3 7RH, UK

[6]Photonics Initiative, Advanced Science Research Center, City University of New York, New York, NY, USA

*These authors contributed equally to the present work

Supplementary Note 1. Tight-binding model calculation

The breathing (expanded or shrunken) honeycomb lattice has 6 sites in a single unit cell and is described (in momentum Bloch space) by the following Hamiltonian, which follows directly from the tight binding model (TBM) [1]:

$$H_0 = \begin{pmatrix} \omega_0 & -\kappa & 0 & -je^{-ik_x} & 0 & -\kappa \\ -\kappa & \omega_0 & -\kappa & 0 & -je^{i(-\frac{1}{2}k_x+\frac{\sqrt{3}}{2}k_y)} & 0 \\ 0 & -\kappa & \omega_0 & -\kappa & 0 & -je^{i(\frac{1}{2}k_x+\frac{\sqrt{3}}{2}k_y)} \\ -je^{ik_x} & 0 & -\kappa & \omega_0 & -\kappa & 0 \\ 0 & -je^{i(\frac{1}{2}k_x-\frac{\sqrt{3}}{2}k_y)} & 0 & -\kappa & \omega_0 & -\kappa \\ -\kappa & 0 & -je^{-i(\frac{1}{2}k_x+\frac{\sqrt{3}}{2}k_y)} & 0 & -\kappa & \omega_0 \end{pmatrix}, \quad (1.1)$$

where ω_0 is on-site energy (frequency), which is set to be zero in what follows, κ and j are intracell and intercell coupling (hopping) coefficients, respectively, and k_x and k_y are the components of the dimensionless (normalized by the lattice constant a_0) Bloch vectors.

For simplicity, in what follows we apply k-p approximation near the Γ-point, where we introduce a unitary transformation operator

$$U_{6\times6} = \frac{1}{\sqrt{6}} \begin{pmatrix} -1 & 1 & -1 & 1 & -1 & 1 \\ 1 & 1 & 1 & 1 & 1 & 1 \\ 1 & e^{i\pi/3} & e^{2i\pi/3} & -1 & e^{-2i\pi/3} & e^{-i\pi/3} \\ 1 & e^{2i\pi/3} & e^{-2i\pi/3} & 1 & e^{2i\pi/3} & e^{-2i\pi/3} \\ 1 & e^{-i\pi/3} & e^{-2i\pi/3} & -1 & e^{2i\pi/3} & e^{i\pi/3} \\ 1 & e^{-2i\pi/3} & e^{2i\pi/3} & 1 & e^{-2i\pi/3} & e^{2i\pi/3} \end{pmatrix}, \quad (1.2)$$

This operator transforms the original Haimltonian breathing honeycomb lattice $H_{cir} = U_{6\times6}H_0U_{6\times6}^{-1}$, and diagonalizes it at the Γ point with the left and right circularly polarized

eigenstates $p_{\pm}$ and $d_{\pm}$, where the p and d letters correspond to the orbital momentum of photon of $l = 1$ (dipole) and $l = 2$ (quadrupole), respectively, and the subscript indicates handedness (pseudo-spin $s = \pm 1$) of the modes.

We then expand the Hilbert space to span the excitonic degrees of freedom with the orbital momentum $m = \pm 1$ at K/K' valleys of the transition metal dichalcogenide (TMD) monolayer, respectively. To this aim the Hamiltonian dimensions are increases by 2 (from 6x6 to 8x8) by adding two exciton states with energies $\omega_+ \equiv \omega_L$, $\omega_- \equiv \omega_R$, whose coupling to photonic degrees of freedom is characterized by the coefficients $q_{p\pm}$, $q_{d\pm}$. Here for simplicity we consider only the case of coupling to the photonic band of interest. We also note that the exciton dispersion can be neglected due the small values of photon wavenumber compared to the exciton wavenumber, and the excitons are always considered as having momentum close to K and K' point in the TMD.

In the case we are discussing below the TR symmetry is preserved, and, therefore, these two excitonic states are always degenerate, $\omega_L = \omega_R = \omega$, and $q_{p(d)+} = q_{p(d)-} = q_{p(d)}$. We thus get an expression of non-dispersive excitonic Hamiltonian ("flat bands"):

$$H_{ex} = \begin{pmatrix} \omega_L & 0 \\ 0 & \omega_R \end{pmatrix} = \omega \begin{pmatrix} 1 & 0 \\ 0 & 1 \end{pmatrix}. \tag{1.3}$$

It is known that these six photonic bands contain six different kind of modes: two singular modes, two dipole modes and two quadrupole modes.

$$H_{8\times8}^{c} = \begin{pmatrix} H_{cir} & Q \\ Q^{\dagger} & H_{ex} \end{pmatrix}, \tag{1.4}$$

$$Q^{\dagger} = \begin{pmatrix} 0 & 0 & q_p & q_d & 0 & 0 \\ 0 & 0 & 0 & 0 & q_p & q_d \end{pmatrix}, \tag{1.5}$$

where the form of Q is dictated by the conservation of the angular momentum $s = m$, that is the photonic of the left $s = 1$ (right $s = -1$) helicity interact only with the excitons at K valley with $m = 1$ (K' valley with $m = -1$) due to the valley polarization (also see Section D below).

The respective transformation matrix then assumes the following form in the extended Hilbert space:

$$U_{8\times8} = \begin{pmatrix} U_{6\times6} & 0 \\ 0 & I_2 \end{pmatrix}, \tag{1.6}$$

Then, in order to reveal the structure of excitons-photon interactions in the original basis, we perform a reverse transformation back to original basis

$$H_{8\times8} = U_{8\times8}^{-1} H_{8\times8}^{c} U_{8\times8} = \begin{pmatrix} H_0 & Q_c \\ Q_c^{\dagger} & H_{ex} \end{pmatrix}, \tag{1.7}$$

and we obtain

$$Q_c^\dagger = \frac{1}{\sqrt{6}} * \begin{pmatrix} q_p + q_d & q_1 + \frac{q_2^2}{q_d} & \frac{q_1^2}{q_p} - q_2 & -q_p + q_d & -q_1 + \frac{q_2^2}{q_d} & -\frac{q_1^2}{q_p} - q_2 \\ q_p + q_d & -\frac{q_1^2}{q_p} - q_2 & -q_1 + \frac{q_2^2}{q_d} & -q_p + q_d & \frac{q_1^2}{q_p} - q_2 & q_1 + \frac{q_2^2}{q_d} \end{pmatrix} \quad (1.8)$$

where $q_1 = (-1)^{\frac{1}{3}} q_p$ and $q_2 = (-1)^{\frac{1}{3}} q_d$. This gives us the form of coupling between the polaritonic bands and photonic bands with this Q_c coupling block.

By introducing interaction of polaritonic bands with the photonic bands in the Dirac cone (without shrinking or expansion) states $p_\pm$ and $d_\pm$, by placing them both mid-gap and to cross with the upper bands, and we calculate the band structure of the resultant system. The respective results are given in Supplementary Fig. 1 and Supplementary Fig. 2.

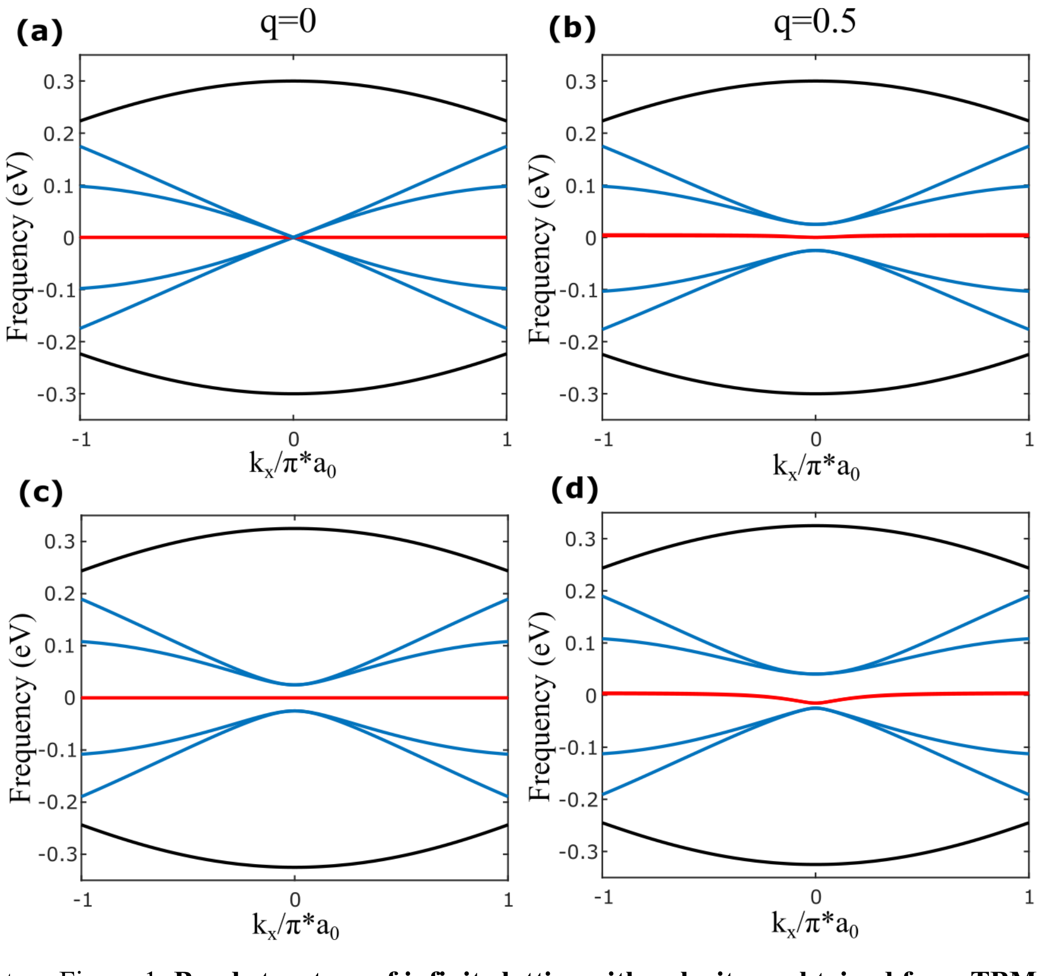

Supplementary Figure 1. **Band structure of infinite lattice with polaritons obtained from TBM model for exciton frequencies exactly at the Dirac point (mid-gap for shrunken/expanded lattice). a** and **b**, unperturbed honeycomb lattice interacting with excitonic bands, q=0 (no interaction) and q=0.5 (with interaction) cases. **c** and **d**, topological (expanded) honeycomb lattice with excitonic states, q=0 (no interaction) and q=0.5 (with interaction) cases.

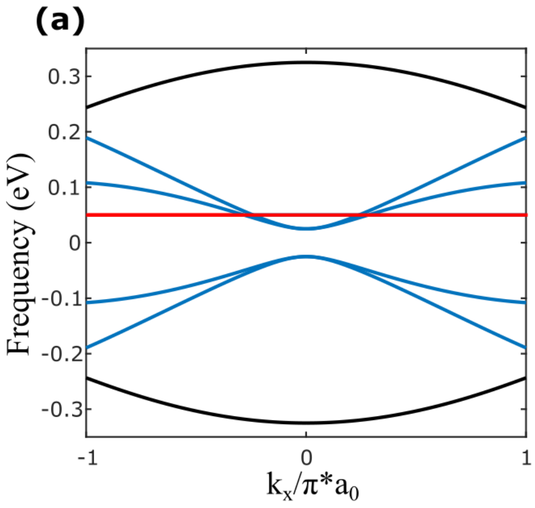

Supplementary Figure 2. **Band structure of infinite lattice with polaritons obtained from TBM for exciton frequencies crossing the upper photonic bands**. **a** and **b**, topological (expanded) photonic honeycomb lattice with excitonic states, for q=0 (no interaction) and q=0.5 (with interaction), respectively.

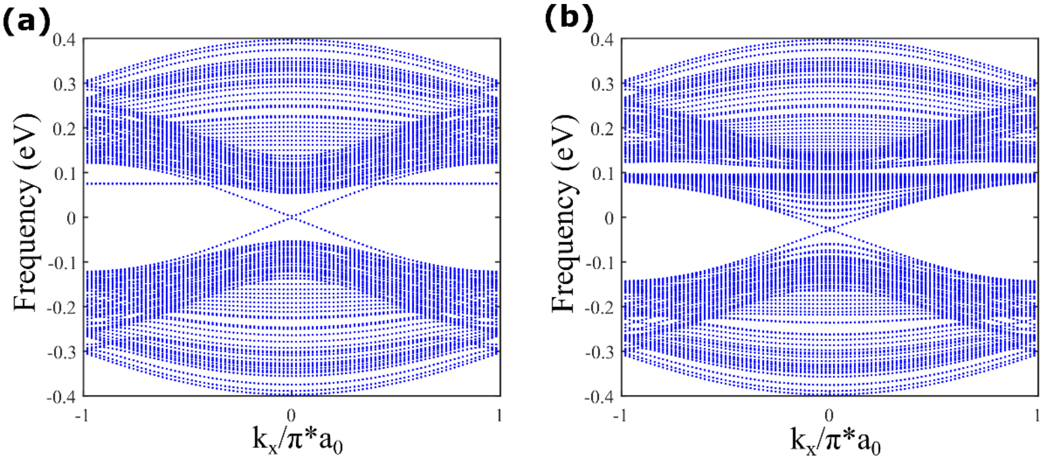

Supplementary Figure 3. **Band structure of a supercell with 10 trivial and 10 topological unit cells with the domain wall in the middle obtained from TBM for exciton frequencies crossing the upper photonic bands**. **a**, no coupling between exciton and photonic bands ($q = 0$), **b**, a strong coupling between polariton bands and photonic bands $q = 1.5$, which is 60% of the average coupling strength between sites $(\kappa + j)/2$.

To observe the emergence of the edge states, we also preformed calculations for the honeycomb supercell consisting of 10 shrunken (trivial) and 10 expanded (topological) unit cells, with the domain wall in the middle. The results in Supplementary Fig. 3 clearly show that the edge states are transferred from photonic bandgap to the gap between the lower photonic bands and the new band corresponding to the exciton-polaritons, while there is no edge state at the upper gap,

indicating the transfer of topological invariant from the former photonic bulk band to the polaritonic band. This is indeed confirmed by the direct calculation of the spin-Chern numbers for the new polaritonic bands, which yields $C_s = \pm 1$ for the two bands.

Supplementary Note 2. Photonic treatment: plane wave expansion near the Γ point and the Berry curvature and spin-Chern number calculations

Without distortion (shrinkage or expansion), the band structure forms two Dirac cones at K and K′. After the distortion is introduced, the Brillouin zone folding occurs, the four-fold degeneracy at the Γ point is lifted and a gap opens. Following Ref. 1, we performed a plane-wave expansion around Γ point and reduced the dimension of resultant matrix to 6x6 by getting rid of the two singlet bands, and then further reduced the dimension of this matric to 3 by 3 matrix by focusing on a particular block corresponding to $s = 1$.

In our system corresponding to experiment, electric field is in-plane, therefore we chose to work with H_z component. The Helmholtz equation for the respective photonic crystal then assumes the following form

$$\nabla \times \left(\frac{1}{\varepsilon(x,y)} \nabla \times\right) H_z + k_0^2 H_z = 0 \quad (2.1)$$

where $k_0 = \frac{\omega}{c}$. In the following equations we replaced k_0 with parameter p.

$$H_z(\mathbf{r}) = \sum_G H_G \, e^{i(\mathbf{G}+\mathbf{k})\cdot\mathbf{r}} \quad (2.2)$$

$$\frac{1}{\varepsilon}(\mathbf{r}) = \sum_G \kappa_G \, e^{i\mathbf{G}\cdot\mathbf{r}} \quad (2.3)$$

$$k_0^2 H_G - \sum_{G'} \kappa_{G-G'} \left[(G_x + k_x)(G'_x + k_x) + (G_y + k_y)(G'_y + k_y)\right] H_{G'} = 0 \quad (2.4)$$

By the integration $\kappa_G = \frac{1}{S_0} \int \frac{1}{\varepsilon(r)} e^{-i\mathbf{G}\cdot\mathbf{r}} \, d\boldsymbol{r}$, we can obtain all orders of refractive index Fourier components needed, S_0 is the unit cell area. $G = \frac{4\pi}{\sqrt{3}a}$ is the length of reciprocal lattice vectors, and we dropped the subscript z for the magnetic field for simplicity as it is redundant.

$$[\kappa_0 \, k^2 - p^2] H_0 + \kappa_1 [k^2 + G \, \mathrm{k_y}] H_1 + \kappa_1 \left[k^2 + G \left(\frac{\sqrt{3}}{2}\mathrm{k_x} + \frac{1}{2}\mathrm{k_y}\right)\right] H_2 + \kappa_1 \left[k^2 + G \left(\frac{\sqrt{3}}{2}\mathrm{k_x} - \frac{1}{2}\mathrm{k_y}\right)\right] H_3 + \kappa_1 (k^2 - G \, \mathrm{k_y}) \, H_4 + \kappa_1 \left[k^2 + G \left(-\frac{\sqrt{3}}{2}\mathrm{k_x} - \frac{1}{2}\mathrm{k_y}\right)\right] H_5 + z\kappa_1 \left[k^2 + G \left(-\frac{\sqrt{3}}{2}\mathrm{k_x} + \frac{1}{2}\mathrm{k_y}\right)\right] H_6 = 0 \quad (2.5)$$

$$\kappa_1 (k^2 + G\mathrm{k_y}) H_0 + \left[-p^2 + \kappa_0 (k^2 + 2G\mathrm{k_y} + G^2)\right] H_1 + \kappa_1 \left[k^2 + G \left(\frac{\sqrt{3}}{2}\mathrm{k_x} + \frac{3}{2}\mathrm{k_y}\right) + \frac{1}{2} G^2\right] H_2 + \kappa_2 \left(k^2 + G \left(\frac{\sqrt{3}}{2}\mathrm{k_x} + \frac{1}{2}\mathrm{k_y}\right) - \frac{1}{2} G^2\right) H_3 + \kappa_3 (k^2 - G^2) H_4 + \kappa_2 \left(k^2 + G \left(-\frac{\sqrt{3}}{2}\mathrm{k_x} + \frac{1}{2}\mathrm{k_y}\right) - \frac{1}{2} G^2\right) H_5 + \kappa_1 \left(k^2 + G \left(-\frac{\sqrt{3}}{2}\mathrm{k_x} + \frac{3}{2}\mathrm{k_y}\right) + \frac{1}{2} G^2\right) H_6 = 0 \quad (2.6)$$

$$\kappa_1\left(k^2+G\left(\frac{\sqrt{3}}{2}\mathrm{k_x}+\frac{1}{2}\mathrm{k_y}\right)\right)H_0+\kappa_1\left(k^2+G\left(\frac{\sqrt{3}}{2}\mathrm{k_x}+\frac{3}{2}\mathrm{k_y}\right)+\frac{1}{2}G^2\right)H_1+\left[-p^2+\kappa_0\left(k^2+2G\left(\frac{\sqrt{3}}{2}\mathrm{k_x}+\frac{1}{2}\mathrm{k_y}\right)+G^2\right)\right]H_2+\kappa_1\left(k^2+\sqrt{3}G\mathrm{k_x}+\frac{1}{2}G^2\right)H_3+\kappa_2\left(k^2+G\left(\frac{\sqrt{3}}{2}\mathrm{k_x}-\frac{1}{2}\mathrm{k_y}\right)-\frac{1}{2}G^2\right)H_4+\kappa_3(k^2-G^2)H_5+\kappa_2\left(k^2+G\mathrm{k_y}-\frac{1}{2}G^2\right)H_6=0 \quad (2.7)$$

$$\kappa_1\left(k^2+G\left(\frac{\sqrt{3}}{2}\mathrm{k_x}-\frac{1}{2}\mathrm{k_y}\right)\right)H_0+\kappa_2\left(k^2+G\left(\frac{\sqrt{3}}{2}\mathrm{k_x}+\frac{1}{2}\mathrm{k_y}\right)-\frac{1}{2}G^2\right)H_1+\kappa_1\left(k^2+\sqrt{3}\,G\,\mathrm{k_x}+\frac{1}{2}G^2\right)H_2+\left[-p^2+\kappa_0\left(k^2+2G\left(\frac{\sqrt{3}}{2}\mathrm{k_x}-\frac{1}{2}\mathrm{k_y}\right)+G^2\right)\right]H_3+\kappa_1\left(k^2+G\left(\frac{\sqrt{3}}{2}\mathrm{k_x}-\frac{3}{2}\mathrm{k_y}\right)+\frac{1}{2}G^2\right)H_4+\kappa_2\left(k^2-G\mathrm{k_y}-\frac{1}{2}G^2\right)H_5+\kappa_3(k^2-G^2)H_6=0 \quad (2.8)$$

$$\kappa_1(k^2-G\mathrm{ky})H_0+\kappa_3(k^2-G^2)H_1+\kappa_2\left(k^2+G\left(\frac{\sqrt{3}}{2}\mathrm{k_x}-\frac{1}{2}\mathrm{k_y}\right)-\frac{1}{2}G^2\right)H_2+\kappa_1\left(k^2+G\left(\frac{\sqrt{3}}{2}\mathrm{k_x}-\frac{3}{2}\mathrm{k_y}\right)+\frac{1}{2}G^2\right)H_3+\left[-p^2+\kappa_0\left(k^2-2G\mathrm{k_y}+G^2\right)\right]H_4+\kappa_1\left(k^2+G\left(-\frac{\sqrt{3}}{2}\mathrm{k_x}-\frac{3}{2}\mathrm{k_y}\right)+\frac{1}{2}G^2\right)H_5+\kappa_2\left(k^2+G\left(-\frac{\sqrt{3}}{2}\mathrm{k_x}-\frac{1}{2}\mathrm{k_y}\right)-\frac{1}{2}G^2\right)H_6=0 \quad (2.9)$$

$$\kappa_1\left(k^2+G\left(-\frac{\sqrt{3}}{2}\mathrm{k_x}-\frac{1}{2}\mathrm{k_y}\right)\right)H_0+\kappa_2\left(k^2+G\left(-\frac{\sqrt{3}}{2}\mathrm{k_x}+\frac{1}{2}\mathrm{k_y}\right)-\frac{1}{2}G^2\right)H_1+\kappa_3(k^2-G^2)H_2+\kappa_2\left(k^2-G\mathrm{k_y}-\frac{1}{2}G^2\right)H_3+\kappa_1\left[k^2+G\left(-\frac{\sqrt{3}}{2}\mathrm{k_x}-\frac{3}{2}\mathrm{k_y}\right)+\frac{1}{2}G^2\right]H_4+\left[-p^2+\kappa_0\left(k^2+2G\left(-\frac{\sqrt{3}}{2}\mathrm{k_x}-\frac{1}{2}\mathrm{k_y}\right)+G^2\right)\right]H_5+\kappa_1\left(k^2-\sqrt{3}G\,\mathrm{k_x}+\frac{1}{2}G^2\right)H_6=0 \quad (2.10)$$

$$\kappa_1\left(k^2+G\left(-\frac{\sqrt{3}}{2}\mathrm{k_x}+\frac{1}{2}\mathrm{k_y}\right)\right)H_0+\kappa_1\left(k^2+G\left(-\frac{\sqrt{3}}{2}\mathrm{k_x}+\frac{3}{2}\mathrm{k_y}\right)+\frac{1}{2}G^2\right)H_1+\kappa_2\left(k^2+G\mathrm{k_y}-\frac{1}{2}G^2\right)H_2+\kappa_3(k^2-G^2)H_3+\kappa_2\left(k^2+G\left(-\frac{\sqrt{3}}{2}\mathrm{k_x}-\frac{1}{2}\mathrm{k_y}\right)-\frac{1}{2}G^2\right)H_4+\kappa_1\left(k^2-G\sqrt{3}\mathrm{k_x}+\frac{1}{2}G^2\right)H_5+\left[-p^2+\kappa_0\left(k^2+2G\left(-\frac{\sqrt{3}}{2}\mathrm{k_x}+\frac{1}{2}\mathrm{k_y}\right)+G^2\right)\right]H_6=0 \quad (2.11).$$

These seven equations establish an effective Hamiltonian for our system.

We then performed a unitary transformation,

$$\widehat{U}_{PW}=\frac{1}{\sqrt{3}}\begin{pmatrix}\sqrt{3} & 0 & 0 & 0 & 0 & 0 & 0\\ 0 & 0 & 1 & 0 & 1 & 0 & 1\\ 0 & 1 & 0 & 1 & 0 & 1 & 0\\ 0 & 0 & 1 & 0 & \mathrm{Exp}[4\pi\, i/3] & 0 & \mathrm{Exp}[2\pi\, i/3]\\ 0 & 0 & 1 & 0 & \mathrm{Exp}[2\pi\, i/3] & 0 & \mathrm{Exp}[4\pi\, i/3]\\ 0 & 1 & 0 & \mathrm{Exp}[4\pi\, i/3] & 0 & \mathrm{Exp}[2\pi\, i/3] & 0\\ 0 & 1 & 0 & \mathrm{Exp}[2\pi\, i/3] & 0 & \mathrm{Exp}[4\pi\, i/3] & 0\end{pmatrix}, \quad (2.12)$$

which block diagonalized this matrix into 3x3 and 4x4 blocks at Gamma point

$$\hat{H}_{3\times3} = \begin{pmatrix} -p^2 & 0 & 0 \\ 0 & -p^2 + G^2(\kappa_0 - \kappa_2) & G^2(\kappa_1 - \kappa_3) \\ 0 & G^2(\kappa_1 - \kappa_3) & -p^2 + G^2(\kappa_0 - \kappa_2) \end{pmatrix}, \quad (2.13)$$

$$\hat{H}_{4\times4} = \begin{pmatrix} h_{1a} & 0 & h_{1b} & 0 \\ 0 & h_{1a} & 0 & h_{1b}^* \\ h_{1b}^* & 0 & h_{1a} & 0 \\ 0 & h_{1b} & 0 & h_{1a} \end{pmatrix}, \quad (2.14)$$

where $h_{1a} = -p^2 + G^2(\kappa_0 + \kappa_2/2)$ and $h_{1b} = (1 + i\sqrt{3})G^2(\kappa_1/2 + \kappa_3)$. The 4x4 block $\mathrm{H}_{4\times4}$ corresponds to the 4 bands forming the Dirac cone.

Addition of k-dependent term obtained from the k dot p theory yield the effective Hamiltonian near Gamma point of the following form (more detail can be found in Supplement, section 2, of Ref. [1])

$$\hat{H}_k = B_2|\mathbf{k}|^2\hat{I} + \begin{pmatrix} u_1 - B(k_x^2 + k_y^2) & 2G(-ik_x + k_y) & 0 & 0 \\ 2G(ik_x + k_y) & u_2 + B(k_x^2 + k_y^2) & 0 & 0 \\ 0 & 0 & u_1 - B(k_x^2 + k_y^2) & 2G(-ik_x - k_y) \\ 0 & 0 & 2G(ik_x - k_y) & u_2 + B(k_x^2 + k_y^2) \end{pmatrix} \quad (2.15)$$

$$u_1 = G^2(\kappa_1 + 2\kappa_3 + 2\kappa_0 + \kappa_2) - 2p^2$$

$$u_2 = G^2(-\kappa_1 - 2\kappa_3 + 2\kappa_0 + \kappa_2) - 2p^2$$

$$B = 2(\kappa_3 - \kappa_1)$$

$$B_2 = 2(\kappa_0 - \kappa_2)$$

This expression is analogous to the Hamiltonian of the BHZ model[2]. We then shift the Dirac cone to zero-energy, rewriting the Hamiltonian in the BHZ form:

$$\hat{H}_k = B_2|\mathbf{k}|^2\hat{I} + \begin{pmatrix} M - B|\mathbf{k}|^2 & A(-ik_x + k_y) & 0 & 0 \\ A(ik_x + k_y) & -M + B|\mathbf{k}|^2 & 0 & 0 \\ 0 & 0 & M - B|\mathbf{k}|^2 & A(-ik_x - k_y) \\ 0 & 0 & A(ik_x - k_y) & -M + B|\mathbf{k}|^2 \end{pmatrix}, \quad (2.16)$$

where $\mathrm{M} = (\mathrm{u}_1 - \mathrm{u}_2)/2$, $A = 2G$, and $|\mathbf{k}|^2 = k_x^2 + k_y^2$.

Then, as in Section A above, we expand the Hamiltonian to include excitons and introduce exciton-photon interactions

$$\hat{H}_D^c = \begin{pmatrix} M - B(k_x^2 + k_y^2) & A(-ik_x + k_y) & 0 & 0 & q_p & 0 \\ A(ik_x + k_y) & -M + B(k_x^2 + k_y^2) & 0 & 0 & q_d & 0 \\ 0 & 0 & M - B(k_x^2 + k_y^2) & A(-ik_x - k_y) & 0 & q_p \\ 0 & 0 & A(ik_x - k_y) & -M + B(k_x^2 + k_y^2) & 0 & q_d \\ q_p & q_d & 0 & 0 & \omega_L & 0 \\ 0 & 0 & q_p & q_d & 0 & \omega_R \end{pmatrix} \quad (2.17).$$

The bands of $\hat{H}_D^c$ are spin-degenerate forming two groups of Dirac cone on top of each other spectrally, therefore we can further focus on the spin-up block establishing the 3x3 Hamiltonian for the respective spin:

$$\hat{H}_D' = \begin{pmatrix} M - B(k_x^2 + k_y^2) & A(-ik_x + k_y) & q_p \\ A(ik_x + k_y) & -M + B(k_x^2 + k_y^2) & q_d \\ q_p & q_d & \omega_L \end{pmatrix} \quad (2.18)$$

We also calculated the Berry curvature and the spin-Chern numbers of each bands of the Hamiltonian given in Supplementary Equation (2.18). Spin-Chern numbers of the band indexed by n is calculated by

$$C^{(n)} = \frac{1}{2\pi} \iint_{BZ} \Omega^{(n)}(\mathbf{k}) \quad (2.19)$$

where Berry curvature $\Omega^{(n)}(\mathbf{k})$ at wave vector $\mathbf{k}$ is $\Omega^{(n)}(\mathbf{k}) = i\nabla_{\mathbf{k}} \times \langle \psi^{(n)}(\mathbf{k}) | \nabla_{\mathbf{k}} \psi^{(n)}(\mathbf{k}) \rangle$. The Berry curvature of the upper polaritonic band ($C^{(2)} = 1$) is shown in Supplementary Fig. 4 and it clearly demonstrated that the dominant contribution arises from the region of avoided crossing, indicating the transfer of the topological charge through the strong coupling mechanism.

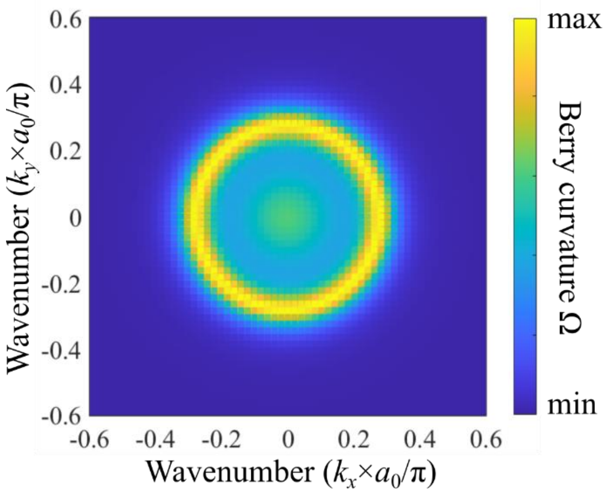

Supplementary Figure 4. **Berry curvature of the polaritonic band**. Berry phase shows a ring-

shaped maximum that appears at the points of avoided crossing, revealing the role of the effective phase winding in the coupling between photons and excitons.

When $u_1 > u_2$ and $B > 0$, or $u_1 < u_2$ and $B < 0$, the system is topological, otherwise it is trivial, yielding zero spin-Chern numbers on any band. We again see that the nontrivial spin-Chern number transfers to the polariton bands. Thus, the spin-Chern number for spin-up states without coupling are {1,0,-1} when the polariton frequency is at midgap and not crossing any band, while after applying a strong coupling with the upper photonic bands, the spin-Chern number for spin-up states transform into {0,1,-1}, and we can see that the nonzero topology invariant has indeed been transferred from upper photonic band to the polaritonic band.

Supplementary Note 3. Circular wave excitation in TBM

To emulate the experimental conditions, a model of coupled mode theory with excitation source in Tight-Binding Model is introduced. In the experiment, a circular polarized source is applied on the whole structure, and measurements of field took place on the boundary of topological and trivial domains, the EM field of a few boundary unit cells are collected.

In TBM model introduced in Section A [Supplementary Equations (1.1), (1.7), and (1.8)], the Hamiltonian of a periodic lattice with excitons $H_{8\times 8}$ contains 8 degrees of freedom, 6 of which are photonic and the other 2 are excitonic. We apply excitation source S_{in} on these 6 photonic bands.

$$S_{in} = \begin{pmatrix} 1 & e^{\pm\frac{i\pi}{3}} & e^{\pm\frac{2i\pi}{3}} & e^{\pm i\pi} & e^{\pm\frac{4i\pi}{3}} & e^{\pm\frac{5i\pi}{3}} & 0 & 0 \end{pmatrix}^{\mathsf{T}}, \quad (3.1)$$

Where plus and minus indicates different field rotation directions, or directions of circular polarizations of light source.

The wavefunction of the system ψ have this relation under excitation

$$\frac{1}{i}\frac{\mathrm{d}\psi}{\mathrm{dt}} = (\hat{H}_{8\times 8} + \mathrm{i}\hat{\gamma})\psi + \alpha S_{in}, \quad (3.2)$$

Where α is the coupling coefficient of the excitation source, and $\hat{\gamma}$ describes is the total loss of the modes of the system. By solving Supplementary Equation (3.2) we can get energy distribution $|\psi|^2$ and reflectivity $r = S_{out} = \alpha\psi$, $R = \alpha^2|\psi|^2$ of the system. Moreover, we can also separate the photonic part and excitonic part in ψ by corresponding index (e.g. in this case, the first 6 elements in ψ is photonic, and the last 2 are excitonic, corresponding to Hamiltonian $H_{8\times 8}$), and the contribution of photonic and excitonic part of the bulk or edge spectrum can thus be shown separately. This method has also been used in supercell Hamiltonian in TBM, where the structure is periodic in one direction, the domain boundaries appear in the other direction. In the main text, by fitting the spectral position and linewidths of spectral lines in experimental data, we estimated the coupling coefficients, Rabi splitting values as well as loss parameters γ_{ph} and γ_{ex} for photonic and excitonic bands, respectively. We also obtained the photonic and excitonic edge state spectra and exciton fractions of the edge state at different frequencies corresponding to experiments in

Fig.4 of the main text.

Supplementary Note 4. Exciton modes coupling evaluation

It's known that excitons in monolayer TMDs are doubly degenerate modes with angular momentum $m = \pm 1$ which represent pairs of electrons and holes at K and K' point, in the atomic Brillion zone, respectively. Consisting of a pair of charged particles, these excitonic states have nonzero dipole moment and can be described as two dipolar modes of opposite helicity. The photonic metasurface in hands, however, possesses more rich structure, with four photonic bands in the frequency range of interest, corresponding to two dipolar $p_{\pm}$ $(l = 1)$ modes and two quadrupolar $d_{\pm}$ $(l = 2)$ modes of opposite helicities $(s = \pm 1)$. It is intuitive to think that the dipolar exitonic modes will couple to the dipolar photonic modes, respectively. However, whether the coupling would happen between exitonic and quadrupolar $d_{\pm}$ modes is not as straightforward because of a completely different spatial scales on which variation of photonic and excitonic wavefunctions occurs, and therefore, it should be carefully analyzed. In this section we aim at finding the coupling between modes various photonic modes and excitonic modes.

The response of a TMD monolayer is homogeneous on the scale of variation of photonic field, and, therefore, it can be expressed by dielectric constant, or, equivalently, by the following surface (sheet) conductivities:

$$\hat{\sigma}_{+} = \frac{\sigma_{TMD}}{2}\begin{pmatrix} 1 & -i \\ i & 1 \end{pmatrix}, \hat{\sigma}_{-} = \frac{\sigma_{TMD}}{2}\begin{pmatrix} 1 & i \\ -i & 1 \end{pmatrix}, \tag{4.1}$$

where σ_{TMD} is the total high-frequency (optical-frequency) surface conductivity of a monolayer, and $\hat{\sigma}_{+}$ and $\hat{\sigma}_{-}$ are contributions to the conductivity from K-valley excitons $(m = +1)$ and K'-valley excitons $(m = -1)$, respectively. The structure of these tensors is dictated by the selection rule on exciton-photon interaction due to the angular momentum conservation, which ensures that the pseudo-spin of photon should equal the angular momentum of excitons $(s = m)$. It is also clear that the cumulative response of the TMD monolayer $\hat{\sigma}_{TMD} = \hat{\sigma}_{+} + \hat{\sigma}_{-} = \sigma_{TMD}$, i.e. isotropic, unless TR symmetry is broken $(\hat{\sigma}_{+} \neq \hat{\sigma}_{-})$.

Note that, because the conductivity tensors $\hat{\sigma}_{\pm}$ do not vary in space, TMD does not mix dipolar and quadrupolar photonic states since they are orthogonal.

We start with the dipolar photonic bands $l = 1$, $s = \pm 1$, which have the following form:

$$|\mathbf{E}_{p+}\rangle = \frac{f(r)}{\sqrt{2}}\begin{pmatrix} 1 \\ i \end{pmatrix}, |\mathbf{E}_{p-}\rangle = \frac{f(r)}{\sqrt{2}}\begin{pmatrix} 1 \\ -i \end{pmatrix}, \tag{4.2}$$

where $f(\mathbf{r})$ describes the field distribution within the photonic unit cell, and $f(\mathbf{r}) \approx 1$ in the weak crystal regime used in the plane wave expansion above. The field vectors are also normalized such that $\langle \mathbf{E}_{ps} | \mathbf{E}_{ps} \rangle = \int dV\, f(\mathbf{r}) = 1$, where integration is over the unit cell volume $(dV = dSdz)$.

The interaction strength between the excitons $(m = \pm 1)$ and the dipolar photonic states $(s = \pm 1)$ can be then calculated as $q_{p(m,s)}^2 = N \int dS_{TMD} \langle \mathbf{E}_{ps} | \mathbf{J}_{pm} \rangle = N \int dS_{TMD} \langle \mathbf{E}_{ps} | \hat{\sigma}_m | \mathbf{E}_{pm} \rangle$, where we used the expression for the excitonic current modes in the TMD monolayer $|\mathbf{J}_{pm}\rangle = \hat{\sigma}_m |\mathbf{E}_{pm}\rangle$

driven by the electric field $|\mathbf{E}_{pm}\rangle$, the integration is over the surface of 2D material in the unit cell, and N is the normalization factor. Then, since the conductivity is homogeneous over the surface, we can readily obtain $q_{d(s,m)} = (N\langle E_s|\hat{\sigma}_m|E_m\rangle)^{\frac{1}{2}} = \sqrt{\frac{N\sigma_{TMD}}{2}}\,\delta_{sm}$. This confirms that the form of the TMD response chosen yields interactions with exciton-photon interactions which respects the conservation of the angular momentum $s = m$, and, consequently, the valley polarization.

Considering the photonic quadrupolar modes ($l = 2$), the field profiles have more elaborate form with the spatial distribution ($d_{\pm} = d_{x^2-y^2} \pm id_{xy}$), where, as before, $\pm$ indicates the photon pseudo-spin $s = \pm 1$, and the field profiles for the case of the weak modulation in the crystal assume the following form in the vector representation:

$$\mathbf{E}_{d+} \sim \begin{pmatrix} x + iy \\ -y + ix \end{pmatrix}, \mathbf{E}_{d-} \sim \begin{pmatrix} x - iy \\ -y - ix \end{pmatrix}. \quad (4.3)$$

Then, the coupling of the excitons and quadrupolar photonic states can be found by evaluating the following expressions: $q_{d(m,s)}^2 = N\int dS_{TMD}\,\langle\mathbf{E}_{ds}|\mathbf{J}_{dm}\rangle = N\int dS_{TMD}\,\langle\mathbf{E}_{ds}|\hat{\sigma}_m|\mathbf{E}_{dm}\rangle$, which can be readily evaluated to yield $q_{d(+,+)}^2 = q_{d(-,-)}^2 \sim \int 2(x^2 + y^2)\,ds \neq 0$, while $q_{d(+,-)} = q_{d(-,+)} = 0$, which again establishes a section rule on interactions between excitons and quadrupolar photons ensuring condition $s = m$. Therefore, the orbital degree of freedom l does not play any significant role in defining selection rules of exciton-photon interactions, which can be interpreted as the result of the orders of magnitude difference in the length scales on which these quantities are defined. Note, however, that the orbital degree of freedom of light l is crucial for the topological photonic phase stemming from the spin-orbit interactions of light.

As we can see here, the two conclusions we arrive at from considerations of this section: (i) there are the spin selection rules for exciton-photon coupling with both dipolar and quadrupolar photonic modes, and (ii) coupling of excitons with quadrupole modes is not equal to zero since the selection rule with respect to the orbital momentum of light does not apply.

Supplementary Note 5. Effective phase winding in exciton-photon interactions

To demonstrate the emergence of phase winding in coupling between photonic and excitonic bands, here we investigate a massive Dirac Hamiltonian interacting with exciton state for one particular spin. The parabolic corrections to the energies (term B in BHZ Hamiltonian) is thus neglected ($B = 0$), which, however, does not affect the generality of our conclusions, since the parabolic correction is known to only change the Chern number by ½ [1], which, as our numerical calculations show, remains true for the more general cases of BHZ and tight-binding models. Thus, we investigate the pseudo-spin-up ($s = +1$) subsystems of photonic system described by the Hamiltonian

$$\hat{H}_{2\times 2}^{+} = \hat{\sigma}_x k_x + \hat{\sigma}_y k_y + M\hat{\sigma}_z = \begin{pmatrix} M & k_x - ik_y \\ k_x + ik_y & -M \end{pmatrix}, \quad (5.1)$$

where $\hat{\sigma}_n$ are Pauli matrices, and Dirac velocity was assumed to be $v_D = 1$ without any loss of generality. The two degrees of freedom in Supplementary Equation (5.1) are implicitly associated with the angular momentum $l = 1$ and $l = 2$ for the case of our system

We can find the eigenmodes of this Hamiltonian for the eigenvalue problem $\hat{H}_+ \bar{\psi}_n^+ = \omega_n \bar{\psi}_n^+$, which have the well-known form

$$\bar{\psi}_1 = \frac{1}{N_1}\begin{pmatrix} 1 \\ f_1 e^{i\theta} \end{pmatrix}, \bar{\psi}_2 = \frac{1}{N_2}\begin{pmatrix} 1 \\ f_2 e^{-i\theta} \end{pmatrix}, \tag{5.2}$$

and the spectrum $\omega_{1(2)} = \pm\sqrt{k^2 + M^2}$, where $k = |\mathbf{k}|$ and $\theta = \tan^{-1}\left(\frac{k_y}{k_x}\right)$, and $f_{1(2)} = -\frac{k}{m-\omega_{1(2)}}$, and $N_{1(2)} = \left|\bar{\psi}_{1(2)}\right|$ is the normalization factor.

Thus, the Hamiltonian Supplementary Equation (5.1) can be diagonalized by the unitary transformation

$$\hat{U}_{2\times2} = [\bar{\psi}_1, \bar{\psi}_2]. \tag{5.3}$$

By expanding the Hamiltonian to include $m = +1$ exciton with the energy $\omega_{ex} = -M$ and exciton-photon interactions $q = q^*$ with the lower band, we obtain

$$\hat{H}_{3\times3}^+ = \begin{pmatrix} M & k_x - ik_y & 0 \\ k_x + ik_y & -M & q \\ 0 & q & -M \end{pmatrix}, \tag{5.4}$$

which can be block diagonalized by applying the expanded unitary transformation

$$\hat{U}_{3\times3} = diag\left[\hat{U}, 1\right], \tag{5.5}$$

yielding

$$\hat{\hat{H}}_{3\times3}^+ = \begin{pmatrix} \omega_1(k) & 0 & q/N_1 \\ 0 & \omega_2(k) & qf_2/N_2 e^{i\theta} \\ q/N_1 & qf_2/N_2 e^{-i\theta} & -M \end{pmatrix}, \tag{5.6}$$

Where one can clearly see the phase to emerge in the coupling between the (diagonalized) photonic eigenstate and the exciton. Then, we consider the fact that the exciton is degenerate at k=0 with the lower (2nd) band and use the degenerate perturbation theory. Therefore, interaction with the upper (1st) band can be neglected giving the effective Hamiltonian

$$H_{2\times2}^{+(eff)} = \begin{pmatrix} \omega_2(k) & \tilde{q}e^{i\theta} \\ \tilde{q}e^{-i\theta} & -M \end{pmatrix}, \tag{5.7}$$

where $\tilde{q}(k) = qf_2/N_2$ is the strength of the effective exciton-photon coupling.

We consider the effect of such interactions near $k = 0$, where the degeneracy takes place, to understand the structure of the eigenmode and energy splitting. In this case the energy ω_2 can be approximated with $\omega_2 = -M - \frac{k^2}{2M}$. Shifting the global energy by $+M$, we then obtain the

eigenvalue problem:

$$\begin{pmatrix} -\frac{k^2}{2M} & \tilde{q}e^{i\theta} \\ \tilde{q}e^{-i\theta} & 0 \end{pmatrix}\tilde{\psi} = \tilde{\omega}\tilde{\psi}, \tag{5.8}$$

which, for $k\sim 0$, gives eigenvalues $\tilde{\omega} \approx -\frac{k^2}{4M} \pm \tilde{q}$, indicating avoided crossing with the splitting of $2\tilde{q}$ near Γ-point, and both eigenstates $\tilde{\psi}\sim[1, \pm e^{-i\theta}]$ become 50% excitonic and 50% photonic.

Supplementary Note 6. Processing of the angle-resolved reflectivity maps

Since the samples were fabricated using SOI substrates, all the measured angle-resolved reflectivity maps possessed characteristic Fabry-Pérot (FP) background, see Supplementary Fig. 5a. This feature hinders the observation of the modes of topological polaritonic system. In order to get rid of this background without affecting the spectral position of the reflectivity features of interest, we resorted to a post-processing procedure. The slowly varying background in the measured map was approximated with a cubic smoothing spline (Supplementary Fig. 5b). The extracted background was then used to calculate the differential reflectivity. This resulted in images with good visibility of the photonic and polaritonic modes of the structure, see Supplementary Fig. 5c and the reflectivity maps throughout the main manuscript. The relevant parameters of polaritonic and photonic modes, however, were extracted from cross-polarized reflectivity and PL maps, which are free from FP background and, consequently, provide the accurate values for spectral position and linewidth of the modes.

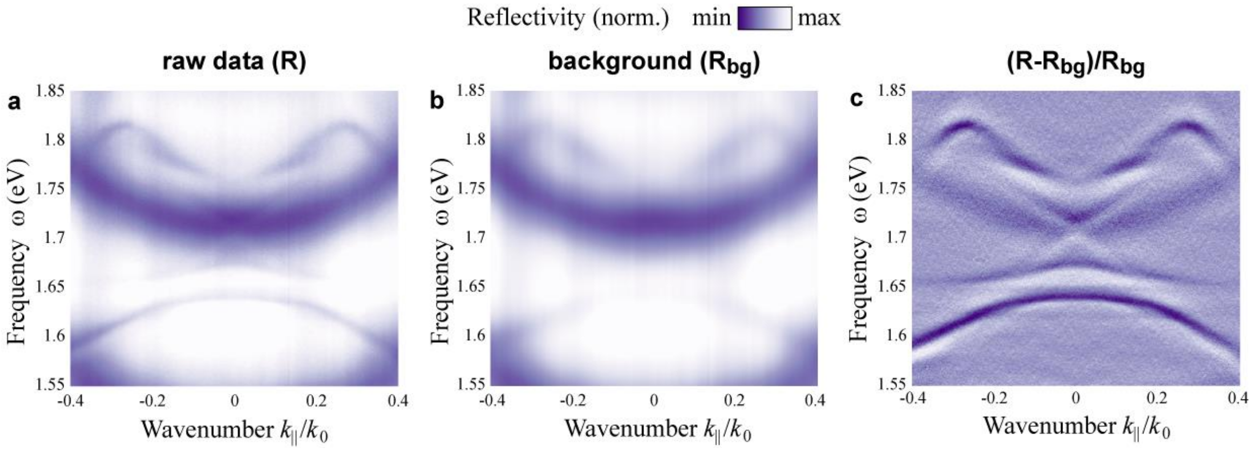

Supplementary Figure 5. **Background subtraction procedure for angle-resolved reflectivity maps. a**, raw reflectivity data for $MoSe_2$ sample. **b**, background approximated with cubic spline. **c**, angle-resolved differential reflectivity map.

Supplementary Note 7. Experiments with a thicker hBN layer

Supplementary Figure 6 shows the angle-resolved differential reflectivity maps for the sample with

60-nm-thick hBN layer between the metasurface and the $MoSe_2$ monolayer. Red shift of the photonic modes driven by increased thickness of hBN leads to the case when the exciton frequency is inside the topological gap of the metasurface and do not exhibit strong coupling with the photonic modes.

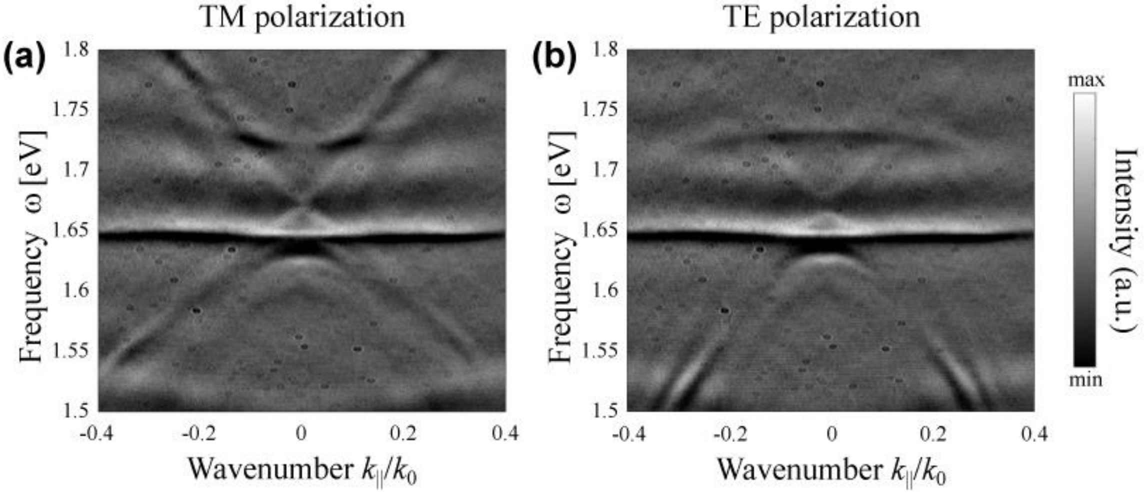

Supplementary Figure 6. **Angle-resolved differential reflectivity of sample with thick hBN layer**. **a**, TM polarized excitation **b**, TE-polarized excitation.

Supplementary Note 8. Temperature tuning and one-way propagation of edge topological polaritons.

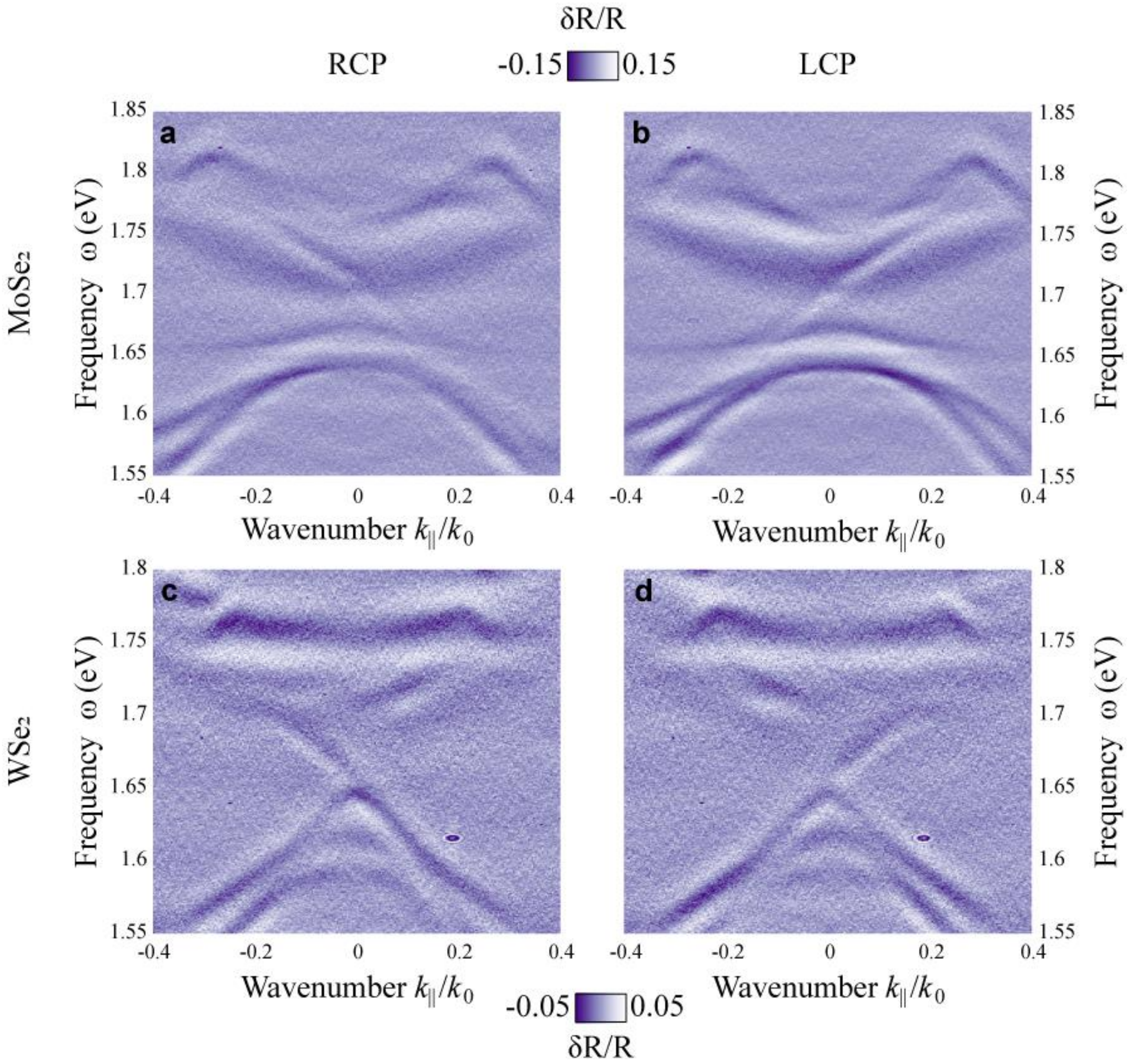

Supplementary Figure 7. **One-way propagation of polaritonic edge states**. **a,b** Angle-resolved differential reflectivity for light with two opposite helicities (**a,** RCP; **b,** LCP) incident on topological metasurface with $MoSe_2$. **c,d** Data for identical experiment with WSe_2 sample.

Topological polaritonic regime can be tuned by shifting the frequency of the exciton resonance *via* changing the ambient temperature. The corresponding results in Supplementary Fig. 8 show the cases of excitation with linearly polarized source at T=7K, 100K, and 200K, which results in the change of the spectral position of exciton with respect to the topological band edge affecting the degree of excitonic component of the edge state. Besides the possibility of tuning, it is notable that, even at relatively high temperatures, the excitons and photons exhibit strong coupling leading to the emergence of a topological polaritonic quantum spin-Hall-like phase. This hints at the

possibility of controlling the degree of valley polarization of topological exciton-polaritons by temperature, which is of great interest for valleytronic applications.

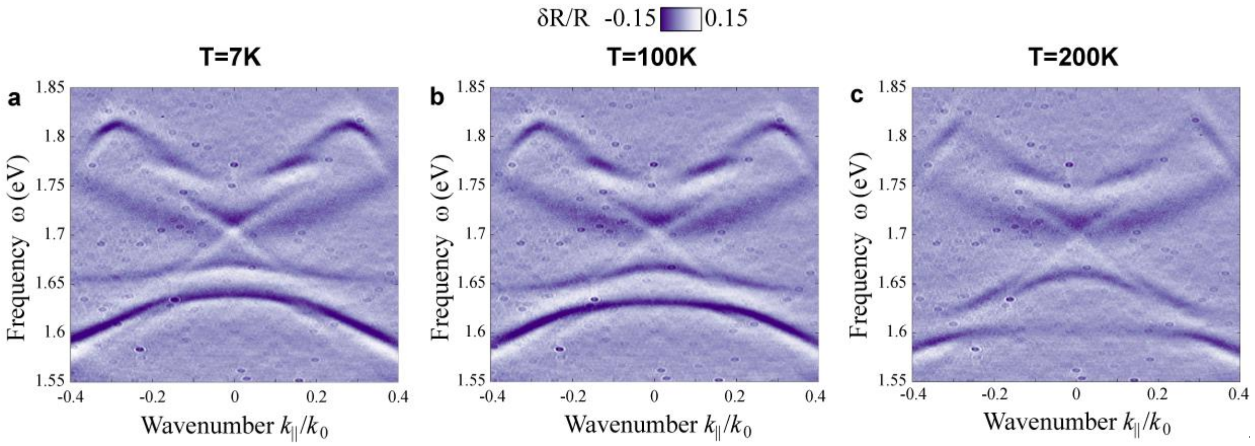

Supplementary Figure 8. **Tuning TPs' dispersion through temperature control over the exciton frequency**. The dispersion of the bulk and edge states extracted from the back focal plane TM polarized differential reflectivity maps at **a** T=7K, corresponding to the exciton frequency of ~1.65 eV, **b** T=100K, corresponding to the exciton frequency of ~1.63 eV, and **c** T=200K, corresponding to the exciton frequency of ~1.61 eV.

Supplementary Note 9. Estimate of the lattice parameters for different TMD monolayer materials

Supplementary Table 1. **Structural parameters for different TMD layers and exciton positions for crossing with either upper or lower photonic bands.**

**Exciton touching with/TMD used**	**$MoSe_2$**	**WSe_2**	**WS_2** (loss in Si can be a problem)
**Upper band**	$a_0 = 488nm$	$a_0 = 440nm$	$a_0 = 360nm$
**Lower band**	$a_0 = 448nm$	$a_0 = 404nm$	$a_0 = 330nm$
**Si device layer thickness** $h = 75nm$			

Supplementary References

1. Gorlach, M.A., Ni, X., Smirnova, D.A. et al. Far-field probing of leaky topological states in all-dielectric metasurfaces. Nat Commun 9, 909 (2018).

2. Bernevig, B. A., Hughes, T. L. & Zhang, S. C. Quantum spin hall effect and topological phase transition in HgTe quantum wells. *Science.* **314**, 1757–1761 (2006).
